# The within host dynamics of *Mycobacterium avium* ssp*. paratuberculosis* infection in cattle: where time and place matter

**DOI:** 10.1186/s13567-015-0185-0

**Published:** 2015-06-19

**Authors:** Ad P Koets, Shigetoshi Eda, Srinand Sreevatsan

**Affiliations:** Department of Bacteriology and TSE, Central Veterinary Institute of Wageningen UR, Lelystad, The Netherlands; Department of Farm Animal Health, Faculty of Veterinary Medicine, Utrecht University, Utrecht, The Netherlands; Department of Forestry, Wildlife and Fisheries, University of Tennessee, Koxville, TN 37996 USA; Veterinary Population Medicine Department, College of Veterinary Medicine, University of Minnesota, St Paul, MN 55108 USA

## Abstract

Johne’s disease or paratuberculosis, caused by *Mycobacterium avium* subsp. *paratuberculosis* (MAP), occurs in domestic and wild animals worldwide, causing a significant economic loss to livestock industries. After a prolonged incubation time, infected cattle shed MAP bacilli into feces and spread the disease to an uninfected animal population. It is largely unknown how (or whether) the interplay between the pathogen and the host immunity determines timing of shedding after the long incubation time. Such information would provide an understanding of pathogenesis in individual animals and the epidemiology of MAP infection in animal populations. In this review, we summarize current knowledge of bovine Johne’s disease pathology, pathogenesis, immunology and genetics. We discuss knowledge gaps that direly need to be addressed to provide a science-based approach to diagnostics and (immuno)prophylaxis. These knowledge gaps are related to anatomical/clinical manifestation of MAP invasion, interaction of bacteria with phagocytes, granuloma formation, shedding, establishment and kinetics of adaptive immune responses in the pathogenesis of the disease. These topics are discussed at the molecular, cellular and tissue levels with special attention to the within host dynamics including the temporal and the spatial context relevant for the various host-pathogen interactions.

## Table of contents

1. Introduction

2. Natural and experimental infection: anatomic manifestation of infection

2.1. Granuloma dynamics

2.2 Bacterial shedding

3. Mononuclear phagocyte - MAP interactions

4. Adaptive immunity during paratuberculosis infection

4.1. Infected macrophage - T cell interaction

4.2. The Th1 – Th2 paradigm revisited

4.3. Immunity in the intestinal wall

5. Within host spatial aspects of MAP infections: targeting immunity to the lesion

5.1. Intestinal compartment

5.2. Mesenteric lymph nodes and blood

5.3. Towards a different dynamic within host model for MAP

6. Conclusions

7. Abbreviations

8. Competing interests

9. Authors’ contributions

10. Acknowledgements

11. References

## 1. Introduction

Paratuberculosis, caused by *Mycobacterium avium* ssp. *paratuberculosis* (MAP), is a chronic intestinal infection of ruminants. Although a small proportion of calves are able to clear the infection, the majority of exposed calves will become chronically infected for life. A fraction (10%) of chronically infected cattle will develop a fatal progressive form of the disease during its life-time. The progressive form of clinical paratuberculosis is characterized by chronic intractable diarrhea in cattle and weight loss, production losses and severe emaciation leading to death since no cure is available. Substantial economic losses to the dairy industry are a result of the infection [1].

MAP infection in dairy cattle occurs predominantly but not exclusively early in life when calves are most susceptible to infection [2]. This susceptibility is associated with the presence of a large number of transient ileal Peyers Patches (PP) in young ruminants providing a large number of M-cells as the major portal of entry in combination with a developing immune system. The presence of infection induced MAP antibodies may enhance the uptake of MAP. During the first year of life these ileal PP go into regression leaving the jejunal PP [3]. In addition to traversing M-cells, MAP is shown to bind and enter through enterocytes due to active MAP - enterocyte interaction [4]. The extent of MAP uptake by M cells was found to be greater than that by enterocytes [5]. Using an in vitro co-culture system, Lamont et al. showed that recruitment of macrophages to the apical side of epithelial cells occurred within 10 min and was dependent on interleukin (IL) 1β produced by the epithelial cells [6]. Following transcytosis MAP is taken up by resident macrophages [3] and dendritic cells (DC) present in the subepithelial lamina propria and remain locally at the site of infection without systemic dissemination [7]. As recently reviewed by Arsenault et al., inside the macrophages and DC, MAP survives and replicates while modulating the intracellular environment of the antigen presenting cell as well as cell surface expression of molecules and the release of cytokines to allow long-term intracellular presence [8].

Johne’s disease is characterized by a long incubation period (1.5–2 years) before cows become fecal culture positive for MAP [9]. Immunodiagnostic tests based on serum antibody responses are generally delayed even more (up to 3 years post exposure) [9]. In experimental settings both antigen specific antibody and T cell responses can be detected within 3–6 months post infection [10]. In approximately 10% of infected cattle clinical signs such as decreased milk production, weight loss, and intermittent diarrhea are observed, typically between 4–6 years of age [11]. In some cattle with pre-clinical and advanced paratuberculosis infection antibody responses predominate and cell mediated responses wane to a point of MAP antigen specific T cell anergy [12]. Based on these patterns derived primarily from cross-sectional studies of Johne’s disease using PBMC and serum based diagnostic tests, it has been hypothesized that progression to clinical disease is a result of a shift from potentially protective cell mediated immune responses to a non-protective antibody response [13,14]. A role for IL-10 producing regulatory and or suppressive cells has been proposed in the pathogenesis of paratuberculosis. This is supported by experimental data in cows in the clinical stage of the disease indicating an increased expression of IL-10 and transforming growth factor (TGF)-β limiting expression of IFN-γ [15]. More recent data support the role of macrophage produced IL-10 in down regulation of Th1/IFN-γ responses and disease progression in MAP infected cattle [16]. However Shu et al. showed a marked upregulation of both pro- and anti-inflammatory cytokines in PBMC and mesenteric lymph node (MLN) lymphocytes of clinical paratuberculosis cows where IL-10 was prominent following PBMC stimulation but IFN-γ was prominent following mesenteric lymph node cell stimulation [17]. In addition, Subharat et al. [18] observed a negative association between IL-10 and disease severity at 15 months post experimental challenge indicating that the immunosuppressive properties of IL-10 may limit infection/inflammation driven tissue damage. A number of conceptual problems regarding our understanding of the pathophysiology of bovine paratuberculosis emerge from the current data. At the herd level it is evident that in herds where the infection is endemic, and highly susceptible neonates are born into an environment in which MAP is abundantly present, persistent infection does not occur in all cows. Although some calves may escape exposure and infection during the first few months of life it has also become apparent from experimental infections that some calves appear resistant to infection. This particular group of apparently resistant animals has not been studied extensively. The fact that most if not all the pathogenesis and immunological responses have been described using infected animals may therefore prevent us from learning about the natural protection against the disease. Furthermore, only a small proportion of all naturally and experimentally infected cattle develop a progressive infection leading to clinical paratuberculosis. In most chronically infected cows the pathogen persists in the presence of an antigen specific cell mediated immune response and specific antibodies. It is unclear why there is an apparent lack of effectiveness of this immune response to eliminate infection in most cows. This is further complicated by large variations in immunological responses between cows as well as within cows over time in both field cases and experimentally infected cattle [19]. Some of this variation can be explained by host genetic factors, MAP genetic factors and strain variation as well as environmental factors such as dose and age of exposure [2,20,21].

This review will focus on recent advances in our understanding of the within host dynamics of bovine paratuberculosis, and identify knowledge gaps, which need to be addressed to further our understanding of the pathogenesis of bovine paratuberculosis within and between cows.

## 2. Natural and experimental infection: anatomic manifestation of infection

Intestinal MAP infection leads to the formation of lesions predominantly in the lamina propria of the small intestine as well as in the draining lymph nodes. Macroscopic signs of infection include thickening of intestinal mucosa leading to a typical corrugated aspect, prominent subserosal lymphatics and enlarged mesenteric and ileocecal lymph nodes. These signs are found in advanced cases of (clinical) paratuberculosis often accompanied with muscle and body fat atrophy. In subclinical cases macroscopic signs are non-specific, subtle or absent [22].

In experimental infections with single (high) dose infections, histopathological examinations have also been performed. Sweeney et al. showed that in a short term experimental MAP infection model in which histopathology was performed on up to 39 tissues per calf 3 to 6 weeks after infection no histological evidence of infection could be found even in MAP culture positive small intestinal tissues [23]. This indicates a low number of MAP per cell and a lack of inflammatory response to the infection. Most data on granuloma formation is derived from adult cattle with established infection and it should be recognized that consequently a bias exists towards susceptible cattle as more resistant cattle and/or the more favorable outcomes of the host-MAP interactions are underrepresented. Early lesions, cellular and molecular responses in such resistant cattle may provide critical missing information on underlying mechanisms of MAP clearance. There is a dire need for such data to understand molecular pathogenesis of JD and implement science based mitigation strategies.

Histopathologically intestinal and lymph node changes are classified as granulomatous lesions.

It is recognized that histological changes vary widely, however, bovine paratuberculosis histopathology differs in at least two ways from bovine and human tuberculosis and human leprosy. First, classically organized Type I tuberculoid lesions present in leprosy and tuberculosis are not observed in bovine paratuberculosis. Second, there is the virtual absence of (polymorphonuclear) granulocytes in bovine paratuberculosis granulomas [24,25]. Thus, the histopathological lesions of bovine paratuberculosis resemble Type II lepromatous rather than Type I tuberculoid lesions as less organized lesions are far more frequently observed.

Two main types of paratuberculosis lesions have been described and classified. Lepromatous-like disease with numerous epithelioid cells containing large numbers of acid-fast organisms are present in the lamina propria and submucosa organized in clusters with no visible Langhans’ giant cells and few lymphocytes. Tuberculoid-like disease with similar disease duration showing few acid-fast organisms with numerous Langhans’-type multinucleated giant cells and somewhat increased numbers of mucosal lymphocytes [22]. Notably clinical signs of disease cannot be uniquely associated with the lepromatous or the tuberculoid type of disease and in both presentations, high numbers of bacilli are shed in the feces [22].

A more detailed histopathologic description aimed at describing lesions in subclinical cases of paratuberculosis was proposed by González et al., where lesions were classified as focal, multifocal, and diffuse lesions [25]. Animals in these investigations originated from farms with endemic paratuberculosis. Animals were, however, not systematically tested for MAP infection prior to examination but only post-hoc based on histopathology. This may account for the relatively higher estimate of infection reported (almost 70% of cows) with overrepresentation of subclinical infection. Focal lesions characterized by the accumulation of 5–30 macrophages with abundant slightly foamy cytoplasm were most prevalent. These lesions were typically present in lymphoid tissue, mostly lymph nodes draining the small intestine but rare in the intestinal wall (<1%). Since these observations were not confirmed for pathogen specificity, the study is prone to misclassification since MAP was only detected by immunohistochemistry and/or ZN staining in less than 9% of the cases with focal lesions. Multifocal lesions were manifested in both the lamina propria and the draining lymph nodes. These lesions tested culture positive for MAP in over 90% of the cases. The main difference between focal and multifocal lesion types observed by Gonzalez et al., was the presence of MAP in the multifocal type and a low burden of MAP in the focal lesion. The cows with diffuse lesions had severe granulomatous enteritis and tested MAP culture positive in 100% of the cases. Within the category of diffuse lesions a distinction was made between diffuse multibacillary (high intracellular burden of MAP in foamy macrophages), diffuse lymphocytic (a rare form (<10%) with few macrophages and predominantly lymphocytic infiltrate) and diffuse intermediate (low intracellular burden of MAP in giant cells and macrophages). The frequency of diffuse intermediate and diffuse multibacillary forms is comparable with 40-50% each in the more advanced cases of disease [26]. The most common forms in subclinical stages of paratuberculosis are the focal and multifocal lesions [25].

Relatively little data are available on the local presence and organization of cells from the innate and adaptive immune system using cell specific markers. In bovine paratuberculosis, pluribacillary lesions of the accumulated macrophages appear to be non-activated as judged by the fact that they lack iNOS expression. Nearby crypt regions showed expression of iNOS but this was unrelated to infection [27].

Koets et al. compared frequencies and absolute counts of lymphocytes present in the ileum, ileum draining lymph nodes and blood in subclinically MAP infected, clinically affected, MAP whole cell vaccinated (cows protected from developing clinical signs but not infection) and healthy controls. Cows with clinical signs of paratuberculosis and progressive multibacillary infection had significantly less CD4^+^ lamina propria lymphocytes, and significantly more Tcr1^+^N12^+^ γδ-T cells in the lamina propria. The subclinically infected, MAP whole cell vaccinated and healthy controls had comparable numbers and frequencies of T cells. The observed disease associated differences were restricted to the lamina propria [12]. Weiss et al. compared the lymphocyte subset distribution in the ileum of healthy and subclinically MAP infected cattle. Ilea of MAP infected cattle contained a higher fraction of macrophages and a lower number of lymphocytes while the frequency of polymorphonuclear leukocyte (PMN) remained the same. Cell frequencies in the spleen were comparable between the 2 groups [28]. Lee et al. also showed an increase in macrophages in the ileum of MAP infected cattle and in addition a decrease in PMN compared to healthy cattle [29]. In infected ileum within the lymphocyte population, there was an increase in the frequency of memory CD4^+^ and CD4^+^CD25^+^ regulatory T cells locally and a lower frequency of activated cells. [28] In this study the CD4^+^CD25^+^ subpopulation was regarded as the regulatory T cell population. In mice and humans only the CD4^hi^CD25^+^FoxP3^+^ T cell subset shows natural suppression not the CD4^+^CD25^+^ subset. In cattle regulatory T cells that show functional suppression are a subset of γδ-T cells. Suppression or anergy were not observed when testing CD4^+^CD25^+^ and CD4^hi^CD25^+^FoxP3^+^ T cell subsets [30]. Hence an increase in regulatory T cells may be present but they may be γδ^+^-rather than CD4^+^ T cells which also match observations of increased frequencies of γδ-T cells in cows with clinical paratuberculosis [12].

### 2.1. Granuloma dynamics

A few studies have addressed the temporal granuloma dynamics in cattle during MAP infection. Most of the pathological changes in bovine paratuberculosis have been described as states or a fixed host response characteristic, rather than part of a continuous dynamic microbe-host interplay [27]. More recently, Kruger et al. studied progression of granuloma in a goat experimental infection model. No continuous progression of lesions (extent and severity) was observed and a high level of variation in types of lesions was observed especially at the end of the study (12 months post infection) [31]. Recent studies on the granuloma dynamics in *M. tuberculosis* infected primates provide compelling data that most lung lesions are probably founded by a single bacterium and reach similar maximum burdens. Despite this observation, the fate of individual lesions varies substantially within the same host as the host sterilizes some lesions even while others progress [32].

While this is a complex concept to evaluate in bovine paratuberculosis due to target tissue, time, host, pathogen and environmental factors, a basic model would be helpful for this knowledge gap. Gonzalez et al. discuss the observation that the focal changes have been observed in very early stages of infection as well as in adult cattle. Although they briefly consider that these may represent initial lesions they dismiss this option based on age-based resistance and favor the view that these focal lesions represent latent persistent infection [25]. This would however imply an extreme longevity of individual latently infected macrophages. Estimations of life-span/turnover of macrophages indicate that in rodent macrophages in the liver, lungs and peritoneal cavity vary between 21 and 42 days in steady state conditions [33-35]. Estimates further vary depending on the site of infection and inflammatory state as it is clear that there is an abundant recruitment of monocytes during infection and inflammation [36]. It may therefore be that focal lesions represent new initial lesions and that the observed histology provides a snapshot of a dynamic situation with transitions between states occurring continuously and in a more or less temporally synchronized fashion. Thus it could be hypothesized that multibacillary can go to paucibacillary if insufficient monocytes can be attracted to the lesion to sustain bacterial replication and/or/as a result of an appropriate protective adaptive immune response. This would imply that multibacillary lesions can be sustained for prolonged periods of time only if sufficient immature macrophages can be attracted to the lesion. The observations of MAP positive debris in lacteal in histopathological studies may be relevant here, as infected macrophages do not have an indefinite lifespan. The release of chemokines and MAP components from dying macrophages may be sufficient inflammatory signals to attract new monocytes to the intestinal tissue. In the lesion, epithelioid cells and newly migrated macrophages can form multi nucleated giant cells (MNGC) [37]. Based on the lifespan estimates of monocytes and macrophages and bacterial replication time the duration of a cycle from focal infection to MNGC scar tissue can be estimated between 1–2 months. Alternatively or superimposed on this dynamic cycle multi- and paucibacillary lesions may represent strain differences between MAP which differ in pathogenicity, fitness or level of being adapted to the host environment since it has been shown that multiple MAP strains circulate in an endemic situation and that one cow can be infected by multiple strains [38]. The apparent states leading to histopathological classification of cows as multi- or paucibacillary responders may be the result of lesions developing with a bias towards either type by immune response driving infection independent factors such as gestation, parturition, negative energy balance or other cow level stressors [39] and host genetics [40].

**Figure 1:**
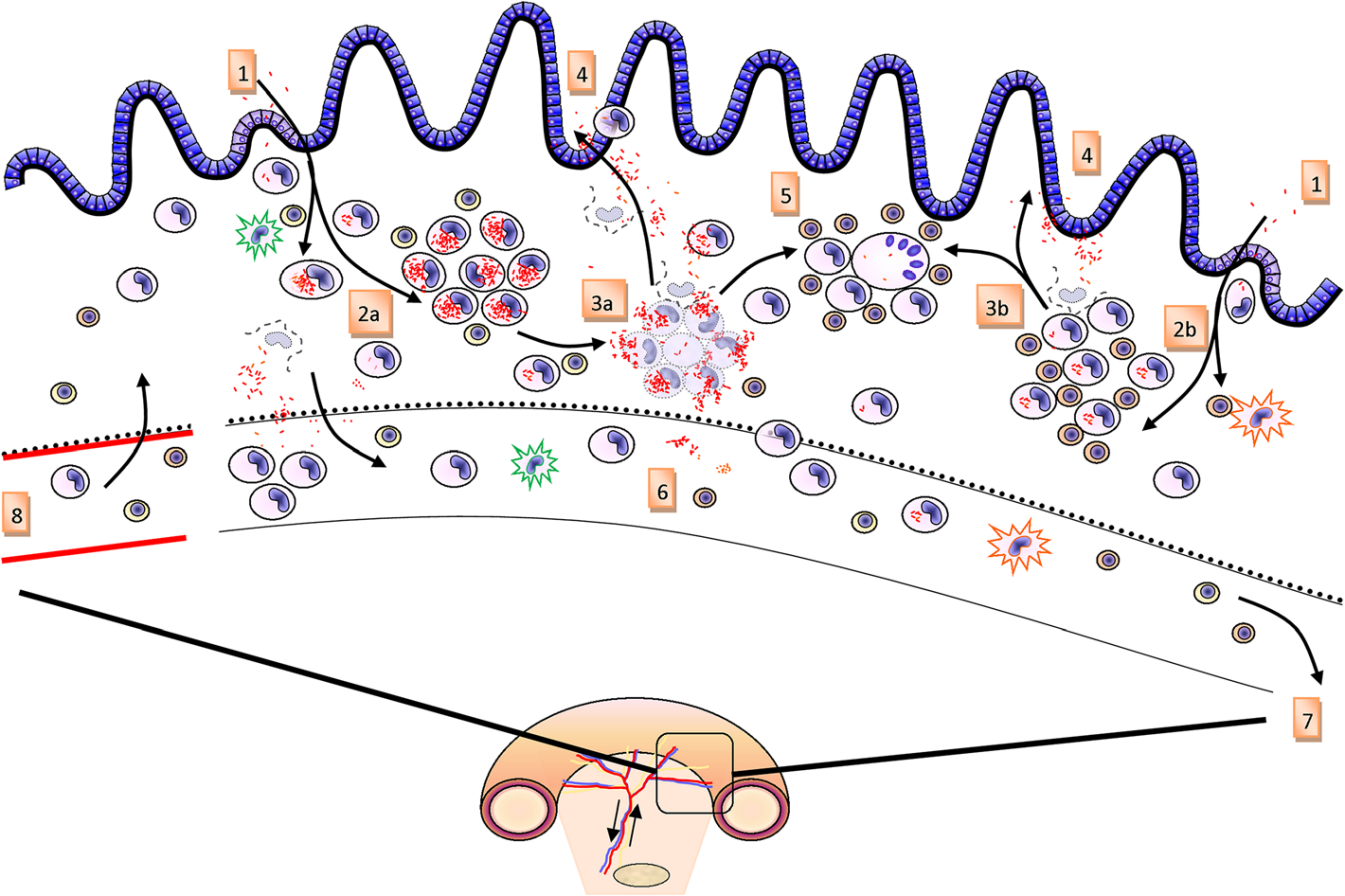
**A model for granuloma dynamics of bovine paratuberculosis.** MAP crosses the intestinal barrier via M cell or enterocyte transcytosis (1) and is subsequently taken up by macrophages in a predominantly tolerizing (2a) or a pro-inflammatory (2b) lamina propria compartment, the state of which may be determined by interplay between different dendritic cells (DC) subsets and enterocytes in combination with antigens present at that particular time. Subsequently the granuloma will develop into a pluribacillary (2a) or a paucibacillary (2b) lesion respectively. Depending on reaching bursting capacity due to bacterial replication (3a) or the end of the natural lifespan of macrophages or non-MAP related causes of cell death (3b) the infected macrophages will die and release MAP and MAP antigens into the lamina propria. Free MAP will enter the intestinal lumen via fluid streams and/or will be taken up by macrophages and DC migrating to the lumen thus leading to shedding of MAP in feces (4). Cellular debris and free MAP antigens from the lesion will be cleared and lead to the formation of scar tissue characterized by multinucleated giant cells and essential devoid of MAP. MAP and MAP antigen taken up by phagocytozing cells residing in the lamina propria may spread to different sites in the intestine and restart formation of a lesion or enter the afferent lymph (6) and migrate to the draining lymph node causing lymph node lesions or activation of T and B cells when taken up and properly processed by antigen presenting cells either on route or in the lymph node (7). Activated T cells and B cell derived antibodies as well as monocytes will enter the intestine via the arterio-venous capillary bed (8).

In a proposed model for the dynamics of bovine paratuberculosis granulomas, focusing on the development of lesions following infection (Figure 1) supposes infectious sources to be extraneous (such as new infections) or intrinsic (such as bacteria released from dying macrophages in an existing infection). Resident macrophages will take up free bacteria rapidly. New infectious loci can subsequently develop into either multibacillary or paucibacillary lesions driven by the disease independent factors described above. Given the limited lifespan of macrophages, we hypothesize cells will die in approximately 4 to 6 weeks and release MAP into the local environment at which point bacteria enter the intestinal lumen and are shed in the feces or remain local and start a new cycle. The remains of the dying macrophages, apoptotic bodies and mycobacterial antigen will be cleared through efferocytosis (ingestion of MAP containing apoptotic bodies by surrounding macrophages) and formation of multinucleated giant cells containing very few if any MAP which will further regress and disappear over time [41].

The high number of focal lesions in the lymph node in early stages of infection may also represent this highly dynamic system of cellular migration [25]. As reviewed by Ehlers and Schaible in murine models of tuberculosis, it has been shown that (initial) granuloma formation progresses independently of the presence of an adaptive immune system [42]. Although care should be taken in translating data from murine models to cattle, a similar observation was made with MAP from bovine origin using severe combined immuno deficient (SCID) mice [43]. Current views on the dynamics of the granuloma are shifting from trying to encompass the heterogeneity of the lesions in a linear temporal setting to a more complex model. In this model Barry et al., discuss how the broad range of responses that occur following TB infection result in the formation of separate microenvironments which can suppress or support bacterial replication, which co-exist in one individual and independently evolve or regress and even disappear over time [44]. Recent experimental data that lesional heterogeneity in *M. tuberculosis* granulomas arises, in part, through differential killing of bacteria after the onset of adaptive immunity. Thus, individual lesions follow diverse and overlapping trajectories, suggesting that critical responses occur at a lesional level to ultimately determine the clinical outcome of infection [32].

### 2.2. Bacterial shedding

Bacterial shedding is commonly used as a diagnostic parameter and a measure for disease activity. Little research has been done to try to elucidate the mechanisms which drive shedding of bacteria and its dynamics. It is important that this process be better understood since the shedding of MAP is a critical step with respect to environmental contamination and transmission of infection.

Despite the fact that diagnosis of infection by fecal culture or fecal PCR is usually performed in adult cattle it has become clear in recent years that MAP shedding does occur in younger animals under field conditions [45]. The frequency of initial shedding in calves younger than 2 years increases substantially with increasing herd prevalence [45]. A recent meta-analysis on the effect of dose and age at exposure also indicates that an early exposure with a high dose is the main driver for early shedding [2]. These data indicate that bacteria are shed in low numbers during early shedding as compared to later stages and likely reflect the progressively expanding granulomatous infection in the intestinal wall.

Several factors may influence the excretion of MAP towards the intestinal lumen. First is that the macrophage, the MAP host cell has a finite lifespan. MAP-infected macrophages may die through apoptosis or necrosis [46]. In apoptosis mediated cell death, plasma membrane integrity is preserved and bacteria are encapsulated in apoptotic bodies. Macrophage suicide through apoptosis enables control of bacterial replication and pathogenic mycobacteria try to prevent the induction of apoptosis in macrophages in which they reside [47]. In MAP infection, apoptosis of infected macrophages is suppressed [48], potentially avoiding clearance through efferocytosis. It is interesting to note that macrophages from Johne’s disease resistant cows are prone to undergo apoptosis [49], suggesting a possibility that enhanced efferocytosis restricts MAP proliferation in animals. However, it has been shown that *Mycobacterium avium* complex (MAC) (strains 101 and 104) either manage to escape from apoptotic bodies to be released in intercellular fluid or survive in apoptotic bodies and upon autophagy of these apoptotic bodies infect other macrophages. The latter process is thought to be an efficient way for MAC to spread to new uninfected macrophages [50].

When prolonged survival occurs, MAP continues replication in the macrophage until the burst size of the macrophages is reached. This leads to macrophage lysis as MAP numbers exceed the macrophages physical limit to further sustain bacterial replication. The burst size for *M. tuberculosis* was estimated to be 20–40 cfu [51]. Based on the observed sizes of (MAP infected) macrophages and an estimated bacterial doubling time in macrophages of 2.8 days [52], burst capacity would be reached within a number of weeks depending on the initial dose. These cells will lyse and a large amount of bacteria and excreted bacterial antigens will be released instantaneously. These MAP will be free in intercellular fluid and can move in the flow of the interstitial fluid. In the fluid, specific antibodies can bind to MAP which may lead to activation of effector mechanisms such as complement [53]. The (opsonized) MAP can be washed out in the interstitial fluid to become lymph and migrate to the draining lymph node. Both ways can lead to dissemination of MAP to other intestinal locations, gut lumen or draining lymph nodes, depending on local biochemical signals [54].

Second, migrating young monocytes, macrophages or DC can phagocytose MAP. MAP is transported to the lumen when infected macrophages and/or DC migrate there. Recent data from studies with surgically isolated ileal segments in calves show that segments remain relatively stable for prolonged periods and significant changes in mucosal leukocyte populations (T cell, macrophage, DC and natural killer (NK) cell) are correlated with the presence or absence of culturable microflora [55]. The presence of intestinal flora is a major factor regarding the presence and migration of monocytes, macrophages and DC based on signals derived from the microflora and its interaction with enterocytes and intra epithelial lymphocytes (IEL) resulting in chemokine production attracting cells [55]. Short term studies with the surgical intestinal segment model indicate that the presence of MAP in the lumen specifically leads to the migration of lamina propria lymphocytes (LPL) and IEL macrophages (and likely DC) into the gut lumen thus proving a potential mechanism for MAP shedding [54]. In vitro macrophage migration studies with MAC-T bovine epithelial cells showed that MAP, through induction of IL1β production and phagosomal acidification when invading the MAC-T epithelial cells, can induce IL1β driven recruitment to and transepithelial migration of monocyte derived macrophages [6]. This mechanism may serve both the recruitment of monocytes to the intestinal epithelium which may take up MAP released from enterocytes but may also provide a model for infected macrophages to reach the intestinal lumen via transepithelial migration.

In the more advanced stages of disease, (partial) obstructions in intestinal lymphatics have been observed, causing enlarged afferent lymphatic vessels. This increases pressure in villus lacteal ducts, which are subsequently dilated and can lead to fistula formation from the lacteal duct to the intestinal lumen. Hence the occurrence of a fluid stream with cells (macrophage, iDC) and MAP is a potential passive mode of translocation of (free or cell associated) MAP to the intestinal lumen and a source of protein loss [22]. Also it has been observed that MAP containing macrophages are present among the glandular epithelial cells, protruding towards the lumen [25]. This migration to the intestinal lumen may also be a driver of shedding especially if the normal flow of lymph is disrupted. Likewise given a highly dynamic monocyte and macrophage migration dynamic in the intestinal wall a single infected cell can migrate to a different site in the lamina propria and start a new initial focal lesion. These mechanisms are captured in Figure 1.

## 3. Mononuclear phagocyte - MAP interactions

The mononuclear phagocytes (macrophages, DC) are the primary target cells for MAP in which it is able to persist and replicate. It has become clear that MAP has extensive abilities to subvert the host innate immune system as has recently been reviewed in detail by Arsenault et al. [8] The initial contact between MAP and the mononuclear phagocytes and the receptors used for uptake are important for the subsequent fate of both MAP and the host cell. Selective uptake via certain receptors such as the integrins, mannose receptor and CD14 influences the macrophage response and may lead to suppression of the oxidative burst, and release of pro-inflammatory cytokines [56]. Opsonization of MAP via FcR (specific antibodies) and CR3 (complement receptor 3, CD11b/CD18) can lead to the induction of oxidative burst, changes in intracellular trafficking and phago-lysosomal acidification leading to reduced survival of MAP. However these effects are critically dependent on prior activation of macrophages by IFN-γ /lipopolysaccharide (LPS). In non-activated macrophages MAP survival and replication is not significantly reduced but rather enhanced [57]. Live MAP, in contrast to dead MAP, also inhibit the phagolysosome fusion by interfering with the endocytic pathway following phagocytosis enabling survival of MAP indicating active evasive mechanisms [58]. Both in macrophages as well as DC [59], infection with live MAP leads to an upregulation of the production of the suppressive cytokine IL-10 and an arrest in mononuclear phagocyte maturation which also renders them refractory to pro-inflammatory signals from activated γδ and CD4 T cells, most notably IFN-γ [60].

The p38-MAPK signaling pathway has been shown to be an important pathway influenced by MAP in bovine macrophages. The p38-MAPK was more rapidly phosphorylated following phagocytosis of MAP by bovine monocytes as compared to *Mycobacterium avium* ssp. *avium* (MAA). These cells which had taken up MAP showed an increase in IL-10 and tumor necrosis factor (TNF)-α expression and a lack of induction of IL-12, lack of acidification of phagolysosomes and reduced MAP killing. Chemical blocking of the p38-MAPK pathway leads to decreased expression of IL-10, increased expression of IL-12 and increased MAP killing indicating that early transient activation of p38-MAPK plays a key role in the capacity of MAP to survive and replicate in macrophages and DC [61]. Transcriptome analysis of infected monocyte derived macrophages indicated that MAP infection of macrophages leads to fast but transient upregulation of genes of the MAPK and IL-10 pathway which have an immunosuppressive effect on adaptive immune responses as well as upregulation of pro-inflammatory genes (IL1B, IL6). Similarly both pro- and anti-apoptotic genes are activated in the early stages of infection. During the first few hours of infection there is an apparent battle between the host and pathogen which wanes from 6 to 24 h post in vitro infection [62].

In conclusion MAP subverts multiple processes in the infected macrophages and DC in the first 6–24 h after infection. These changes which can be summarized as follows: MAP keeps the host cell alive by preventing apoptotic suicide of macrophages; MAP avoids being killed by interfering with phagolysosomal function of macrophages; MAP avoids being detected locally by manipulation of the expression of (signaling pathways of) cytokine and chemokine genes and thereby inducing an immunosuppressive environment and a spatial separation between the intestinal lesion and the systemic immune system; and the intracellular infection of (intestinal) DC leads to a delay or absence of proper DC maturation.

## 4. Adaptive immunity during paratuberculosis infection

### 4.1. Infected macrophage - T cell interaction

The poorly activated or subverted MAP infected DC will neither migrate efficiently to the draining lymph node nor function as a proper pro-inflammatory antigen presenting cell. Since the DC – T cell interaction in the draining lymph node is critical to proper induction of the adaptive immune response this will impair optimal induction of pro-inflammatory protective T cells in the draining lymph node and subsequent migratory properties of these T cells. The consequence being a delayed activation of T cell responses hampering protective immunity due to late arrival at infected sites [42,63]. In addition, changes in the infected macrophage are established rapidly and the basic changes happen in the first 6 – 24 h of infection. If these infected macrophages are trapped in intestinal tissue and do not signal infection and or inflammation they are hard to find for circulating T cells. This leaves a very small window for an effective adaptive T cell based immune response.

In order for the T cell adaptive immune response to be able to stimulate infected macrophages to kill the intracellular mycobacteria (predominantly through CD4 T cells) or to kill infected macrophages cells have to be able to migrate from the blood to a lesion. Upon intracellular infection, the macrophage will through a pro-inflammatory response attempt to attract T cells, however MAP tries to counteract this via the induction of anti-inflammatory pathways but also specifically through inhibition of RANTES and MCP chemokine gene expression in infected macrophages which will result in a lack of T cell recruitment to infection sites [64].

For T cells, which through local inflammatory signals have migrated to the lesions, recognition of infected macrophages critically depends on the specific interaction of the T cell receptor (TCR) with macrophage expressed major histocompatibility complex (MHC) containing MAP specific peptide molecules. However in vitro studies indicated that upon intracellular infection of macrophages and DC, MAP avoids being detected by down regulation of MHC and/or co-receptors of Ag presentation and activation [65,66]. In in vitro generated MAP-infected DC, antigen presentation through MHC class II was impaired [59] and, in MAP-infected cattle, expression of MHC class I molecules were up-regulated at early MAP infection, suggesting a CD8 biased antigen presentation profile [65].

In in vitro infection models after an initial activation of gene expression, MAP rapidly (within 6-24 h) shuts down the pro-inflammatory T cell immunity by induction of immunosuppressive cytokines (IL-10, TGF-β), impairment of CD40 signaling which is an important macrophage receptor for CD40L on Th1 type T cells to maintain a Th1 immune response [66]. Furthermore it has been observed that MAP infection of monocyte derived macrophages inhibits IFN-γ induced signaling. Since NK and T cell derived IFN-γ is thought to be an important cytokine in the protection against intracellular pathogens including pathogenic mycobacteria, inhibition of these pathways is an important virulence mechanism [67].

Finally similar to other pathogenic mycobacterial infections such as tuberculosis, infected macrophages can inhibit or kill adaptive immune T cells through a number of different routes such as contact via Fas/FasL interaction, soluble modulators originating from host cells (TGF-β, TNF-α, FasL and Bcl-2) [68], and secreted bacterial antigens such as in tuberculosis, where the early secreted antigen ESAT-6 has been shown to directly inhibit human T cell responses [69]. The observation that this process may be focused on or biased towards antigen specific T cells contributes to the hypothesis that killing specific T cells is part of the pathogenic mechanism of mycobacteria contributing to the observed late stage T cell anergy. In paratuberculosis this antigen specific CD4 T cell depletion has been observed in particular when studying local intestinal T cell responses [12]. The induction of T cell apoptosis as a result of T cell – macrophage interaction in ovine MAP infection has also been described [70].

### 4.2. The Th1 – Th2 paradigm revisited

Paratuberculosis and other chronic mycobacterial infections typically show persistence of the pathogen in the presence of an antigen specific immune response. For decades, studies have investigated the attributes of a protective immune response. The Th1/Th2 paradigm for classifying CD4 T helper cells has been a cornerstone of functional dissection of adaptive immune responses since the 1980’s when these two lineages of helper T cells were first described by Mosmann et al. [71]. Subsequent studies with anti-mycobacterial responses in genetically modified mice pointed at a pivotal role for IFN-γ / Th1 responses in containing infection as opposed by Th2 / IL-4 mediated responses [72]. Following suit using T cell proliferation and/or IFN-γ and antibody production and/or IL-4 as proxy for Th1 and Th2, respectively the changes during progressive paratuberculosis have been attributed to a shift from an early Th1 biased potentially protective response to a Th2 biased response [14]. Comparisons of subclinically infected cows with cows in advanced clinical stages of infection showed that on average cell mediated responses in PBMC were higher in subclinical cows and antibody levels were higher in clinical cows. Association between bacterial shedding and switch pattern followed the hypothesis that if Th1 is protective then the loss of cell mediated immunity should lead to increased bacterial replication and shedding [11]. The switch has been attributed to infection load, T cell exhaustion and several more generic triggers such as hormonal changes in the periparturient period due to (metabolic) stress. Ultimately it is still unclear what is driving the shift in this model. A recent mathematical modeling study on the Th1/Th2 paradigm indicated that initial dose and bursting size control the timing of the switch [73]. Most of the data underlying these models were derived from PBMC and serum derived from animals in cross sectional studies usually in a setting of optimizing diagnosis of paratuberculosis.

In recent decades the Th1/Th2 paradigm has expanded to much higher complexity by the discovery that naïve CD4 T cells can additionally differentiate into several regulatory T cells (natural T cells with a regulatory phenotype (Treg), inducible Treg), pro-inflammatory Th17 T cells, Th9 T cells and polyfunctional T cells in a complex cross-regulatory network with antigen presenting cells such as DC and macrophages [74].

A role of natural or antigen specific regulatory T cells has been suggested to be involved in the progression of bovine paratuberculosis either directly or as a source of immunosuppressive IL-10 [28,75]. To definitively prove that classical CD4^+^CD25^hi^ T cells are the regulatory T cells driving the switch in bovine paratuberculosis may prove difficult due to the fact that functional suppression in cattle appears to be mediated by macrophages and subsets of γδT cells rather than natural CD4^+^CD25^hi^ Treg as observed in mice and humans [30,75]. In addition, most of these pathogenesis studies rely on in vitro assays and blood derived lymphocytes. Antigen specific functional data with intestinal lymphocytes is scarce although data gained with intestinal lymphocyte preparations do point to a lack of functional Th1 type T cells [12,28]. These studies show a clear difference between functional data obtained with PBMC, MLN cells and lamina propria lymphocytes and add to the question to which extent the circulating blood lymphocytes are representative of the local intestinal immunity.

In long-term longitudinal follow-up studies of (mostly) experimental infections it has, however, been difficult to confirm this pattern of switching from early cell mediated responses to late antibody responses. Especially from the longitudinal studies it has become apparent that both Th1 (IFN-γ) and Th2 (IgG1 antibody) responses occur, also in early stages of disease without clear indication of associating to progression and clinical disease [10,76]. In addition early transient induction of IL-10 as well as B cells have been observed following experimental infection in conjunction with IFN-γ as an early marker of infection [77,78].

There is an altered pattern of TLR gene expression in culled cows naturally infected with MAP. The data suggests that the upregulation of the expression of TLR-1 in response to MAP infection appears to be impaired in MLN and PBMC from infected cattle upon stimulation with MAP antigen [79]. Using the same cells it was reported that MAP antigen stimulation of MLN cells from the severely infected group with high lesion scores leads to significant upregulation of the mRNA expression of IFN-γ, IL-10, IL-13, IL-17A, and TNF-α. There was no significant upregulation of these cytokines in the control and less severely infected groups. In addition, major differences were observed between the responses of the PBMC and MLN cultures. Higher levels of secreted IFN-γ from the MAP stimulated MLN cultures and, conversely, higher levels of IL-10 are released from the PBMC cultures [17]. Other studies using direct ex-vivo analysis of cytokine gene expression indicated an upregulation of IL-10 and TGF-β expression and a down regulation of IFN-γ expression in the intestinal wall when comparing subclinical and clinical cases of paratuberculosis [80]. Together these data show clear differences in response patterns from lymphocytes isolated from varying anatomical locations, notably MLN and blood. In addition, the data indicated that differences may be (in part) related to the antigenic load in the infected animal, which differs at different stages of disease. A recent histopathological study in combination with immune response data from Vazquez et al. indicated that when comparing lesion severity and adaptive immune responses, the IFN-γ and antibody responses appear to be better correlated with overall lesion severity and bacterial load rather than indicating a switch from cell mediated (Th1) to Th2 responses [26]. Recent views in the field of tuberculosis also indicate that IFN-γ is a pivotal cytokine for protection in murine models but in humans and cattle it is simultaneously considered as a marker for bacterial load and disease activity and it may be rather difficult to use as a measure of protection or immunopathogenesis [81]. This has led to the question whether a switch from Th1 to Th2 actually exists in paratuberculosis or whether in progressive paratuberculosis (antigen specific) exhaustion leads to a generalized failure of adaptive immunity in which the Th1 response generally fails first [19].

### 4.3. Immunity in the intestinal wall

It has become clear that the antigen presenting cells (APC) and the local environment where interaction between DC and T cell occurs are critical in determining the differentiation fate of naïve helper T cells and ensuing homing and effector functions [82]. In that respect the gut mucosal localization of the first contact between MAP and the immune system is significant. In the acute stage of controlled infection in a ligated loop infected model resulted in an acute migration of macrophages from the lamina propria into the gut lumen as well as invasion of MAP through epithelial surfaces [54]. After one hour, co-localization of MAP and macrophages in the lumen was observed. At 4 h post infection, infiltration of PMN and mononuclear cells into the lamina propria was observed. The infiltration of PMN and mononuclear cells was progressive during the first 12 h of infection [54]. Histopathological examination does not permit specific detection of infection at the early stages (1–12 weeks) after oral infection and changes such as the accumulation of PMN and mononuclear cells in the distal ileum are not observed [23]. This may signify a sampling and sensitivity issue, however, recent data also point to an early induction of host immune tolerance pathways by MAP during the first 12 h of infection and the abrogation of PMN and mononuclear cell migration thus a part of the pathogenic mechanisms enabling MAP survival and persistence [83].

Adequate pro-inflammatory response following uptake by the macrophages in the intestinal tract is especially challenging since the intestinal environment has to balance tolerogenic mechanisms to prevent adverse reactions to innocuous food components while maintaining an adequate defense against food borne pathogens [84]. The distal ileum in young ruminants is in essence a continuous PP which will regress during the first year of life almost exclusively occupied by B cells. There is a massive outflow of B cells from the anatomic site. In the early months of life the terminal ileum is exempt from the normal T cell recirculation indicating that T cell mediated immunity is non-existent in the area [85].

Recent experimental infections using an intestinal segment model in 10–14 day old calves studied immune response at 1 month and 9–11 months post infection [86,87]. Responses to early infection show that in the infected intestinal segments diffuse aggregates of acid fast bacteria are present in the lumen but no acid fast organisms can be observed following ZN staining in the mucosa or submucosa. All tissue samples of infected segments were, however, PCR positive indicating the presence of MAP DNA in the (sub)mucosa. Analysis on adaptive immune responses at this stage shows that in four calves, two responded with a serum antibody response to a 35 kD antigen while the others showed no antibody response to this antigen. None of the calves showed a clear IFN-γ response in PBMC except for one antibody responder. Neither of the two antibody responders showed a high IFN-γ response with cells from the draining MLN while both the calves that were seronegative had high MLN responses. Therefore these four calves were evenly split into two cell-mediated immunity (CMI) responders and two antibody responders. A parallel 10 segment per calf kinome response analysis also indicated a dichotomy, which upon comparison mirrored the immune response dichotomy. Pathway and gene ontology analysis revealed that differences in innate immune and interleukin signaling and particular differences in the Wnt/β-catenin pathway distinguished the kinomic groupings [87]. Apparently very early after infection, differences in adaptive immune response patterns can be seen. Furthermore it is clear that MLN and PBMC responses are clearly different within individual animals. It is also clear that since all tissues were MAP PCR positive neither type of adaptive response pattern is protective. Finally it should be noted that two of three control calves also showed an IFN-γ response to MAP lysate. This may indicate a more innate type of response directly to the MAP lysate possibly through NK cell activation or sensitization to environmental mycobacteria. In a similar experiment from the same group these ileal segments were examined nine months post infection. They showed that MAP was localized in the intestinal segment (sub)mucosa and was not detected by PCR in the mesenteric lymph node. MAP specific CD4 and γδ − T cell responses were observed in MLN. In the lamina propria numbers of CD8 and γδ-T cells increased and MAP-specific TNF-α and IFN-γ secretion by lamina propria leukocytes was also increased. There was a significant accumulation of macrophages and DC in the lamina propria, but the expression of mucosal TLR one through ten was not significantly changed by MAP infection [86]. Interestingly no increase in lamina propria CD4^+^ T cells was observed despite antigen specific CD4 T cell induction in MLN indicating a potential problem with the lymphocyte recirculation to tissue lesions. Again these responses did not confer protection to chronic infection, as MAP PCR of tissue was consistently positive with a lack of ZN staining indicating that the number of bacilli was small. Plattner et al. recently described studies with a matrigel skin implantation model and show that matrigel loaded with dead MAP leads to influx of CD4^+^ T cells. However in placebo control cows and cows with matrigel containing live MAP, no migration of CD4^+^ T cells is observed indicating that live MAP prevents macrophages and DC from either the induction of T effector memory cells and or generating the right signals to attract these CD4^+^ T cells to the site of infection [88]. Although this model is a skin based model it points out a potential mechanism leading to an inability to recruit an effective CD4^+^ T cell response during (early) infection to the site of infection.

## 5. Within host spatial aspects of MAP infections: targeting immunity to the lesion

Current data lead to a number of questions indicating knowledge gaps. Is Th1 the protective response or a measure of severity of disease? Are the peripheral Th1 T cells unable to “home” or find their target when they do home to the (vast) lamina propria)? Is there a bias towards a Th1 response in the early stages of infection?

Initiation of adaptive immune response requires migration of DC properly loaded with antigen and a pro-inflammatory make rather than tolerogenic to the draining lymph node where naïve T cells can be activated. Mucosal surfaces, such as the respiratory tract and the gastro-intestinal tract are part of the mucosal immune system. Taken together the mucosal epithelia comprise a vast and vulnerable barrier, which have to combine exchange of gasses and intake of nutritional components while simultaneously preventing microbial invasion.

Interactions between MAP and the bovine immune system occur at two distinct spatially separated locations connected by the circulatory system. The first compartment is the (small) intestines where lesions (defined as one or more infected macrophages) will form. The intestines and in particular the lamina propria is the primary place for the interaction of MAP with macrophages. A continuous afferent lymph fluid stream provides a unidirectional connection between the lamina propria with the draining lymph nodes. In this second compartment the interaction between the antigen presenting cell antibodies, T cells and B cells takes place. Following the interaction between the antigen presenting cell antibodies, T cells and B cells egress from the draining lymph node and enter the venous circulation. Most immunological and immunodiagnostic observations are made using serum and/or lymphocytes derived from blood which can be seen as a third compartment in this system [84].

Although the number of studies pertaining to MAP infection simultaneously comparing immune cellular composition and functionality is limited, there are clear indications that these compartments contain functionally different immune effector cells. Both phenotypic composition of cells isolated from the blood, MLN and lamina propria as well as antigen specific responses from these isolated cell populations show significant differences based on location as well as stage of infection: the latter pointing to a local loss of CD4^+^ T cells Th1 like proliferative responses and an accumulation of potentially regulatory γδ − T cells [12]. Similarly in young experimentally infected calves, a dichotomy in MAP-specific immune responses was observed when comparing mucosal and systemic responses. To determine if an antibody response to MAP proteins could be detected, using serum samples collected before and one month after infection. Sera from two animals reacted antigen specifically after infection. PBMC isolated from the same two animals showed significant proliferation and IFN-γ responses to MAP lysate. Calves that lacked antibodies reactive to the 35 kDa protein one month after infection showed strong proliferation and IFN-γ responses by MLN cells, but not in PBMC [87].

### 5.1. Intestinal compartment

**Figure 2:**
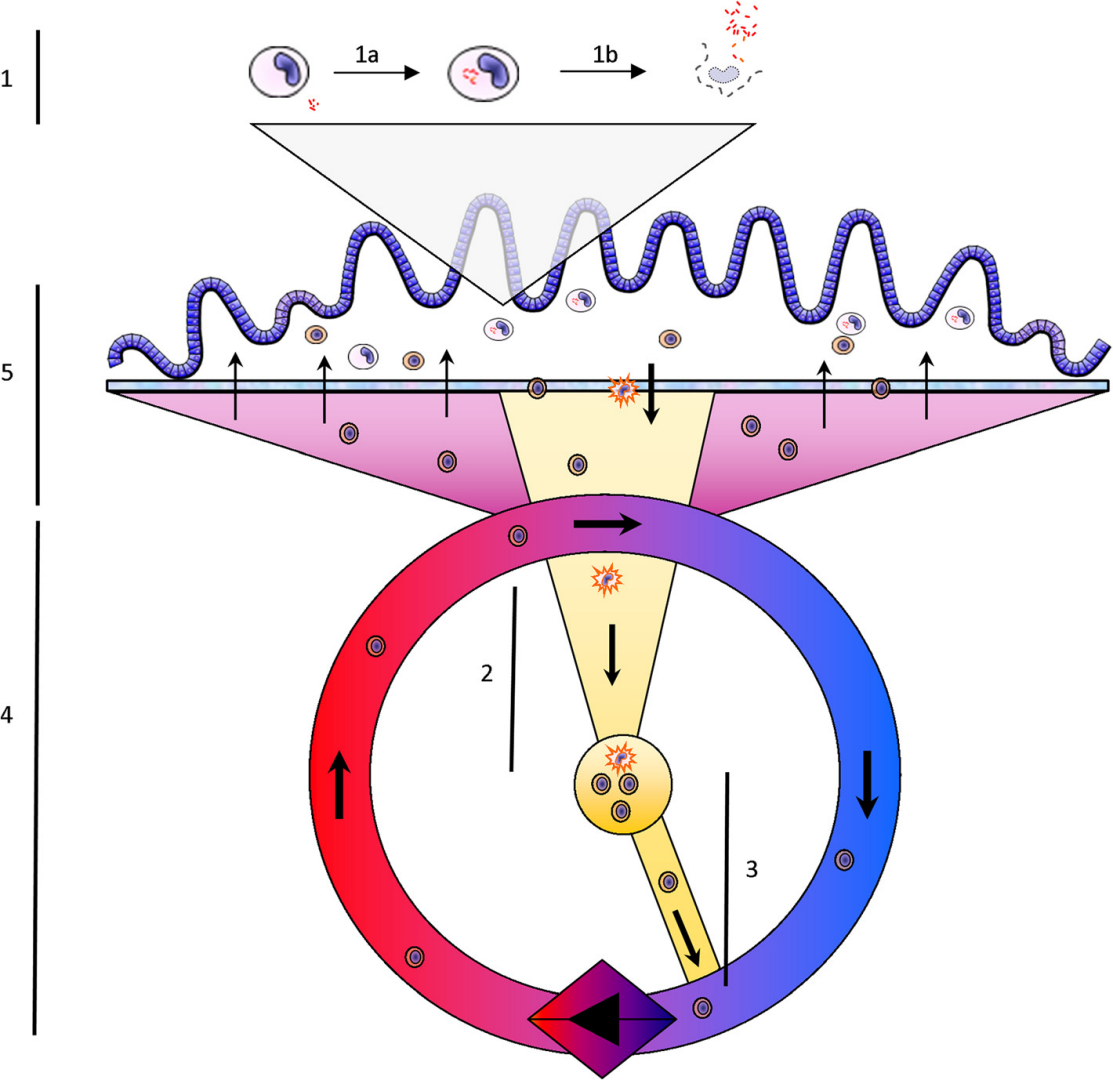
**Spatio-temporal aspects of bovine paratuberculosis.** 1. A. Following uptake of MAP, macrophages will be subverted within 12–24 h into immune suppressed niche environments for bacterial replication. B. Bacterial replication will be limited by the natural lifespan of the macrophage (21–42 days), and/or reaching bursting capacity due to space limitations of harboring dividing MAP bacteria. (1 CFU infection with bacterial replication time of 40 h. will lead to accumulation of 300–500 MAP in 35–42 days). 2. Immature dendritic cells (DC) which have taken up MAP/antigen migrate to the draining mesenteric lymph node. Maturation to professional antigen presenting DC occurs during transit, barring interference by live MAP. 3. In the mesenteric lymph nodes DC will become stationary in the T cell zone to be interrogated by migrating T cells. Antigen specific recognition leads to the induction of effector T cells and clonal expansion, obtain addressins for targeted migration to the intestine and migrate out of the lymph node into the venous circulation over the course of days. 4. The activated and memory T cells will recirculate randomly through the organs and may remain in the circulation for prolonged periods of time (days) depending on their route of migration and additional signals. 5. Passing through arterio-venous capillary beds in the intestine effector T cells may migrate from the circulation into the lamina propria based on their homing receptors. This process may be random when pro-inflammatory chemokine signals are lacking but highly targeted when these signals are present and efficiently directing T cell migration. In the lamina propria these T cells may encounter MAP infected macrophages and start antigen specific effector functions such as IFN-γ production and induction of apoptosis in infected cells. However if the infected macrophage has been transformed to an immunosuppressive state T cell recognition will likely be hampered as will subsequent effector mechanisms.

The intestinal mucosal immune system including the gut-associated lymphoid tissue (GALT) is phenotypically and functionally different from other compartments such as the blood, skin or respiratory tract. This is caused by the fact that in contrast to most other tissues the small and large intestine has to down regulate the continuous physiological inflammation due to the extensive constant antigenic load from luminal microorganisms and pathogen associated molecular patterns (PAMP) to maintain homeostasis. Naïve T and B cells enter the GALT via high endothelial venules similar to entry into lymph nodes. CD4^+^ helper T cells activated in GALT are known to release cytokines such as TGF-β and IL-10, which drive the class switch and differentiation of mucosal B cells to predominantly IgA-committed plasma blasts. Both naïve and primed T and B cells migrate rapidly from GALT via draining lymphatics to MLN where they may be further stimulated; they next reach thoracic duct lymph and peripheral blood to become seeded by preferential homing mechanisms into distant mucosal effector sites (Figures 1 and 2). This process is directed by the rapidly acquired profile of adhesion molecules and chemokines expressed on the intestinal endothelial cells which function as a local gatekeeper for cellular entry into the dominant effector site, the lamina propria. This is modulated by additional signals from local antigen-sampling DC, resident lamina propria CD4+ T cells, and the cytokine milieu. The combined effect of oral tolerance mechanisms, mainly the action of regulatory T cells, provides a suppressive tone in the gut, normally keeping Th2 inflammation driven by IgG and IgE antibodies as well as Th1 cell-mediated responses and delayed-type hypersensitivity (DTH) as well as pro-inflammatory Th17 responses under tight control [82].

MAP is translocated from the intestinal lumen via M cells [3] and enterocytes [5] into the lamina propria and taken up by resident DC and macrophages. Recent data using experimental infection ligated intestinal loops also show migration of macrophages and DC towards the intestinal lumen containing MAP [54]. These data indicate that active sampling of intestinal content by DC may also lead to the uptake of MAP. Recent data suggest that the resident intestinal DC are “educated” by intestinal epithelial cells to suppress inflammation and to promote immunological tolerance. Recent studies in cattle have also indicated the presence of a large variety of intestinal phenotypically different and likely specialized DC with migratory properties [89]. Since MAP enters via M-cell and enterocytes without perceivable tissue damage, there is likely no pro-inflammatory response following the entry of MAP. Subsequently MAP survives inside macrophages and is able to replicate and transform the macrophage/DC into a tolerogenic state. In the early stages of infection there is not much activity in the lamina propria such as attraction of lymphocytes or additional monocytes. Although in (experimental) oral infections it is difficult to find histological lesions, MAP IS900 PCR of tissue is frequently positive and, the targeted intestinal loop infections indicate the existence of a large number of MAP positive macrophages in early stages some of which (will) form granulomas.

Initial stages will therefore be limited to the interaction between MAP within the resident DC/macrophages and local T cells. A commonly used model for the direct MAP-macrophage interactions is the in vitro culture of macrophages (mostly monocyte derived macrophages) with MAP. Caveats in our knowledge are the role of the intestinal microenvironment, time, host and pathogen genetic effects. Functional studies indicate that among the changes occurring in infected macrophages a number lead to death of (Th1) T cells either through cell-cell contact such as Fas/FasL mediated activation induced cell death (AICD) [90] macrophage production of TNFα or the production of T cell toxic molecules excreted by the intracellular mycobacteria [69]. Local data are not abundant, however, in established lesions of paratuberculosis, the T cell population in the lamina propria changes showing a significant loss of CD4^+^ T cells and the concomitant increase of potentially regulatory γδ − T cells [12] indicating that similar mechanisms may occur in MAP granulomas.

Rather than directly originating from lesions it is most likely that early events of MAP specific adaptive immunity are related to uptake of MAP by pro-inflammatory DC or macrophages activated through unrelated events and migrating towards secondary lymphoid organs at the time of encountering MAP or MAP antigens (secreted antigens or remnants of dead mycobacteria). When taken up and processed by pro-inflammatory macrophages proper antigen degradation and presentation will follow in a draining lymph node. This will lead to activation of pro-inflammatory T cells which will enter the circulation. These are typically the antibodies and cells present in routinely-taken samples of peripheral blood [87].

The intestinal afferent lymph contains a large and diverse population of cells. Among these are the recirculating T cells exiting intestinal tissue and migrating towards the draining lymph node. The afferent lymph also contains many immature DC migrating from intestinal tissue to the draining lymph node with antigen [91]. These can be both tolerogenic and inflammatory and ensuing contact with T cells in the lymph node will determine the adaptive response to the presented antigens biasing towards IL-5/IL13 producing Th2 T cells and subsequent B cell activation and antibody production, Th1/Th17 T cells and subsequent generation of IFN-γ/IL17 producing pro-inflammatory effector T cells and/or IL-10 producing Treg with anti-inflammatory properties. In general intestinal baseline T cell cytokine signatures are biased towards a Th2 and/or Treg T cells rather than Th1 [92].

### 5.2. Mesenteric lymph nodes and blood

The induction of effector T cells requires the movement of antigen-loaded APC to a secondary lymphoid organ (e.g. mesenteric lymph node) and the presentation of antigen to specific T cells. Upon activation these T cells need to commit and differentiate into effector and memory T cells. Subsequently these cells have to exit the lymph node, enter the circulation and migrate to the intestine. Although data is rather scarce especially in cattle it has been reported using models of efferent and pseudo-afferent lymph cannulation that it would take the γδ − T cell population abundantly present in pseudo-afferent lymph 46 h for a full surveillance of the drained skin area [93]. When studying vaccination induced activation of CD4 T cells in the lymph cannulation model using MAP Hsp70 as antigen it takes 2 days for antigen specific T cells to start exiting from the lymph node to enter the circulation [94]. So both the time to activate effector cells as well as the time the system requires to survey the peripheral tissue well exceeds the 12–24 h MAP needs to impose its immune evasion mechanisms on the macrophage following macrophage infection. However upon return to the intestine, chances are in overwhelming favor of the situation that the activating antigen cannot be found and the responder T cells will recirculate without seeing their antigen in the proper context during their lifespan.

As a consequence even with the generation of an in theory protective IFN-γ^+^ CD4^+^ T cell response the chance of failure of these T cells to detect infected macrophages is substantial (see Figure 2).

The majority of the T cells that are found when sampling venous blood in infected animals will reflect the amount of antigen arriving at the various intestinal draining lymph nodes and the context in which they are locally presented in the lymph node to T cells. As such they may reflect the intestinal disease activity and circulation of free antigen between the intestinal wall and the draining lymph node rather than be a measure of protective or permissive immune responses. In that sense the responses measured using PBMC could be used to predict disease outcome but should be interpreted with great care with respect to pathogenesis and correlates of protection since PBMC responses may not be representative of local reactions [95]. This notion is enhanced by a surprising lack of agreement between cytokine responses obtained from antigen stimulated PBMC and MLN cells from naturally infected cattle [17]. Earlier data comparing PBMC, MLN and LPL antigen stimulated recall responses also indicated this discrepancy [12]. In a study in which CD4^+^ T cells were depleted in infected calves using monoclonal antibodies, no effect on the course of disease was observed despite documented T cell depletion [96]. Finally also T cell immunosuppressive treatment of latent phase MAP infected cows does not accelerate disease progression [97]. These data point to an inability of the adaptive T cell response to mount a protective immune response at the infection sites. This is likely a combination of the time it takes to mount a T cell response, get T cells to the lesion and the more rapidly acquired immunosuppressive status of locally infected macrophages to among others block T cell effector mechanisms.

### 5.3. Towards a different dynamic within host model for MAP

The overall spatio-temperal aspect of the pathogenesis of bovine paratuberculosis has not been comprehensively studied thus far. Current data has made it clear that MAP has an elaborate array of immune evasive mechanisms and the ability to change infected macrophages into niches for replication in a matter of 6–24 h. From vaccination studies we know that it takes days to weeks to generate an adaptive immune response which can be measured in blood samples. However major efforts aiming at the development of vaccines inducing Th1 type immune responses have not yielded major successes in either tuberculosis or paratuberculosis.

The temporal aspects of the development of immune responses as observed in peripheral blood samples has been studied in detail, however, the variation in response patterns seems to preclude a uniform pattern. The kinetics of T cell responses thought to be protective are elusive. A re-evaluation of the role of CD4^+^ T cells is especially needed. With evidence building that CD4^+^ T cells are unable to locally perform their task, and a lack of efficient homing to the lesions observed, responses in peripheral blood derived CD4^+^ T cells made constitute an epiphenomena related to the severity of infection rather than a measure of control over the infection. This also suggests that the course of infection that we observe regarding the MAP shedding pattern is independent of the adaptive immune responses measured.

The role of B cells and antibodies has been largely ignored in MAP and TB, however, mouse studies have shown antibody mediated protection in TB models [98]. Antigen specific antibody responses more consistently appear 2–3 years after infection. As a philosophical question it can posed whether the “lack” of antibody response in early stages of mycobacterial infection is coincidental or is there an active immune evasion induced by MAP and other pathogenic mycobacteria to prevent such induction of B cell responses which is then even more pronounced than the effect on T cell responses. Although early activation of B cells has been documented, this appears to be abrogated as a potential pathogenic mechanism of MAP [78]. Vaccine induced antibody mediated protection with Hsp70 vaccination in cattle has been shown both when applied early after infection [99] as well as in an extreme post exposure setting [100]. As antibodies are homogenously dispersed in serum and interstitial fluid there are no migration issues as with T cells. As a result of a limited life span of macrophages MAP has to leave the macrophage periodically to find new host cells. This provides opportunities for antibody MAP interaction and thus provides multiple intervention points. The ultimate challenge lies in identifying early “infectious phenotype” associated antigens of MAP which can be used as a vaccine target for the prevention of new infection. In addition, its combination with antigens such as the 70 kD heat shock protein (Hsp70) would benefit infection control as well. So in reality, a multipronged approach is needed.

## 6. Conclusions

MAP is a highly successful intracellular pathogen that has specialized mechanisms of effacement, entry and establishment of infection in a wide range of hosts. Existing experimental and natural infection derived data on immune responses clearly indicate a complex, yet a deterministic pattern. The applications of immune responses and modulation of cellular populations in the infected tissues as well as in circulation as biomarkers of protection against a vaccine or establishment of infection and progression of disease still need refinement. Furthermore, MAP genomic variations and their in-vivo correlations with immune response modulation or interaction with hosts of a variety of genetic backgrounds is not clearly established. Focused longitudinal studies on MAP progression of disease as modulated by host or pathogen genotype are needed. With evidence mounting that a strong (vaccine induced) Th1 type T cell mediated immunity does not lead to protection per se and indications that vaccine induced antibodies may contribute to protection in tuberculosis and paratuberculosis the reevaluation of the dynamic aspects of the pathogenesis of paratuberculosis is necessary since it may aid conceptual thinking of the concept of protective immunity.

## 7. Abbreviations

MAP: *Mycobacterium avium* ssp. *paratuberculosis*
PP: Peyer’s patch
IL: Interleukin
DC: Dendritic cell
IFN: Interferon
PBMC: Peripheral blood mononuclear cell
IGRA: Interferon Gamma Release Assay
TGF: Transforming growth factor
MLN: Mesenteric lymph node
TB: Tuberculosis
ZN: Ziehl–Neelsen
iNOS: Inducible nitric oxide synthase
KO: Knock-out
PMN: Polymorphonuclear leukocyte
MNGC: Multi nucleated giant cells
SCID: Severe combined immuno deficient
MAC: *Mycobacterium avium* complex
NK: Natural killer
IEL: Intra epithelial lymphocytes
LPL: Lamina propria lymphocytes
TLR: Toll-like receptor
CR: Complement receptor
LPS: Lipopolysaccharide
MAA: *Mycobacterium avium* ssp. *avium*
TNF: Tumor necrosis factor
TCR: T cell receptor
MHC: Major histocompatibility complex
Treg: T cells with regulatory phenotype
APC: Antigen-presenting cell
CMI: Cell-mediated immunity
GALT: Gut-associated lymphoid tissue
PAMP: Pathogen-associated molecular pattern
AICD: Activation induced cell death
